# Commensal *Streptococcus agalactiae* isolated from patients seen at University Hospital of Londrina, Paraná, Brazil: capsular types, genotyping, antimicrobial susceptibility and virulence determinants

**DOI:** 10.1186/1471-2180-13-297

**Published:** 2013-12-21

**Authors:** Eliane Saori Otaguiri, Ana Elisa Belotto Morguette, Eliandro Reis Tavares, Pollyanna Myrella Capela dos Santos, Alexandre Tadachi Morey, Juscélio Donizete Cardoso, Márcia Regina Eches Perugini, Lucy Megumi Yamauchi, Sueli Fumie Yamada-Ogatta

**Affiliations:** 1 Laboratório de Biologia Molecular de Microrganismos, Departamento de Microbiologia, Centro de Ciências Biológicas, Universidade Estadual de Londrina, Rodovia Celso Garcia Cid, PR 445, km 380, Londrina, Paraná, CEP 86057-970, Brazil; 2 Laboratório de Microbiologia Clínica, Departamento de Patologia, Análises Clínicas e Toxicológicas, Centro de Ciências da Saúde, Universidade Estadual de Londrina, Londrina, Paraná, Brazil

**Keywords:** Group B *Streptococcus*, Capsular antigen, Erythromycin, Clindamycin, MLVA typing, *pilus* island, Virulence traits

## Abstract

**Background:**

*Streptococcus agalactiae* or Group B Streptococci (GBS) have the ability to access various host sites, which reflects its adaptability to different environments during the course of infection. This adaptation is due to the expression of virulence factors that are involved with survival, invasion and bacterial persistence in the host. This study aimed to characterize GBS isolates from women of reproductive age seen at University Hospital of Londrina, according to capsular typing, genetic relatedness, antimicrobial susceptibility profile and occurrence of virulence determinants.

**Results:**

A total of 83 GBS isolates were enrolled in this study. Capsular types Ia (42.2%), II (10.8%), III (14.5%) and V (30.1%) were identified in most GBS. One isolate each was classified as type IX and non-typeable.

A total of 15 multiple *locus* variable number of tandem repeat analysis (MLVA) types were identified among the isolates, seven were singletons and eight were represented by more than four isolates. All isolates were susceptible to penicillin, ampicillin, cefepime, cefotaxime, chloramphenicol, levofloxacin and vancomycin. Resistance to erythromycin and clindamycin was observed in 19.3 and 13.3% of isolates, respectively. All isolates resistant to clindamycin were simultaneously resistant to erythromycin and were distributed in the capsular types III and V. One isolate showed the constitutive macrolide-lincosamide-streptogramin B (cMLS_B_) phenotype and ten showed the inducible MLS_B_ (iMLS_B_) phenotype. The mechanism of resistance to erythromycin and clindamycin more prevalent among these isolates was mediated by the gene *ermA,* alone or in combination with the gene *ermB*. The isolates displaying resistance only to erythromycin belonged to capsular type Ia, and showed the M phenotype, which was mediated by the *mefA/E* gene. All isolates harbored the gene *hylB* and at least one *pilus* variant, PI-1, PI-2a or PI-2b. Although *cylE* was observed in all GBS, four isolates were classified as gamma-hemolytic and carotenoid pigment non-producers.

**Conclusions:**

Our results indicate the potential virulence of commensal GBS isolates, reinforcing the need for continued screening for this bacterium to prevent infections. The distribution of capsular and *pili* antigens, and MLVA profiles was also identified, which may contribute to the development of new strategies for the prevention and treatment of GBS infection.

## Background

*Streptococcus agalactiae* (Group B Streptococci – GBS) can colonize the gastrointestinal and genitourinary tracts of healthy individuals without any symptoms of disease [1]. Nevertheless, this bacterium can cause life-threatening invasive diseases in pregnant women, neonates or non-pregnant adults. Colonized women, during pregnancy or the postpartum period, are usually asymptomatic, but GBS may cause bacteremia, urinary tract infections, chorioamnionitis, endometritis, puerperal sepsis and, occasionally meningitis and septic thrombophlebitis [2,3]. GBS colonization among pregnant women also increases the risk of premature delivery and perinatal transmission of the microorganism to newborns, which can cause fatal sepsis and meningitis [4,5]. A successful perinatal disease prevention strategy based on intrapartum chemoprophylaxis for pregnant women at risk [6] leads to a significant decrease in GBS infections in neonates [3,6,7]. However, in the last decades, GBS have been increasingly associated with invasive disease in non-pregnant adults, mainly in the elderly, immunocompromised and those with diabetes mellitus and cancer [3,8,9]. Most importantly, mortality associated with these patients is frequently higher than for newborns [3,8]. These data draw attention to the need for prevention strategies against GBS infections among adults.

Penicillin has been established as a first-line antimicrobial for the prophylaxis and treatment of GBS infections. Moreover, clindamycin and erythromycin have been used as alternatives in penicillin-allergic individuals. However, resistance to these antimicrobials among GBS isolated from pregnant and non-pregnant individuals has been described in several countries [3,9-15], raising concerns about their use in the treatment of GBS infections. Resistance to penicillin is frequently associated with mutation of penicillin-binding proteins (PBP) 2X and 2B [14]. Overall, the mechanisms that confer resistance to erythromycin include the post-transcriptional methylation of the adenine residues of 23S ribosomal RNA mediated by *erm* genes and efflux of the antibiotic mediated by a membrane-bound protein encoded by *mef* genes. The expression of *erm* genes results in the MLS_B_ phenotype, responsible for generating cross-resistance to macrolides, lincosamides and streptogramin B [16]. On the other hand, phenotype M, encoded by *mef* genes, confers resistance only to 14- and 15-membered ring macrolides (erythromycin and azithromycin) [17].

According to the immunologic reactivity of sialic acid-rich capsular polysaccharide, GBS are divided into ten serotypes, Ia, Ib, II-VIII [18] and IX [19]. Different surveys all over the world have shown the prevalence of serotypes Ia, Ib, II, III and V as major streptococcal disease-causing agents [3,7-9,20,21].

The diverse array of clinical manifestations caused by GBS reflects an efficient adaptability of bacteria to different host environments. GBS may express virulence factors, allowing the colonization and invasion of epithelial barriers, leading to resistance to immune clearance and persistence in host tissues, which contribute to the pathogenesis of infection. Besides defining GBS serotypes, the cell wall-anchored polysaccharide capsule has been recognized as important virulence factor of this bacterium. It prevents the deposition of alternative complement pathway factor C3b on the bacterial surface, resulting in decreased phagocytosis by macrophages and neutrophils [22]. In the last decade, a *pilus*-like structure was identified in GBS [23] and shown to play an important role in the adhesion to and invasion of host cells [24], biofilm formation [25] and resistance to phagocyte killing [26]. Extracellular β-hemolysin/cytolysin (β-H/C) is a pore-forming toxin encoded by the chromosomal *cylE* gene [27], which is toxic to a broad range of eukaryotic cells, resulting in cell invasion [28] and evasion of phagocytosis [29]. The expression of *cylE* is also associated with the production of an orange carotenoid pigment that contributes to the protection of bacteria against the toxic effects of reactive oxygen species generated by the oxidative burst mechanism of phagocytic killing of macrophages [29]. The product of gene *hylB*, a secreted hyaluronate lyase, can hydrolyze hyaluronan polymers, which are components of the extracellular matrix of human tissues, suggesting that this enzyme can facilitate the spread of bacteria during infection [30].

In the study described here, GBS isolated from women at reproductive age with no clinical evidence of streptococcal infection were characterized by phenotypic and molecular methods. All isolates were tested for capsular type, hemolysis and carotenoid pigment production. In addition, the *in vitro* susceptibility pattern of the isolates to antimicrobial agents, the genetic relatedness and the occurrence of virulence determinant genes were also investigated.

## Results

### Patients, GBS capsular types and multiple *locus* variable number of tandem repeat analysis (MLVA) genotypes

**Figure 1:**
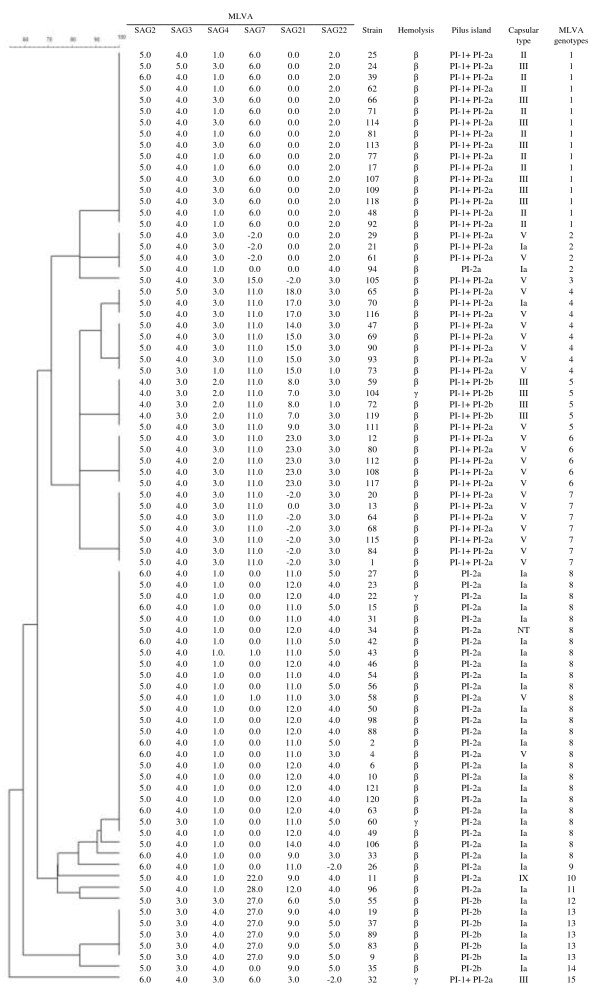
**Phenotypic and genotypic features of 83 commensal *Streptococcus agalactiae *isolated from patients seen at University Hospital of Londrina**: **distribution of capsular types, *pili *island, hemolysis pattern and MLVA types.** Cluster analysis was performed using UPGMA algorithm of the Bionumerics v. 4.6 software, with a cutoff value set at 85%. Numbers of repeats are showed in each MLVA marker. The number -2.0 was assigned if no PCR product could be amplified. Hemolysis in agar plate containing 5% sheep blood.

A total of 83 isolates of GBS from women with no clinical evidence of streptococcal infection were enrolled in this study. These isolates were taken from the bacterial collection of the Laboratory of Clinical Microbiology of University Hospital of Londrina, the major referral center for healthcare management that serves Londrina city, besides several localities of Paraná, São Paulo and Mato Grosso do Sul states, in Brazil. The age of the patients ranged from 15 to 58 years (median 29.7 years old). GBS isolates were distributed in five capsular types according to the multiplex-PCR method, and type Ia (35/83, 42.2%) was the most frequent, followed by type V (25/83, 30.1%), type III (12/83, 14.5%) and type II (9/83, 10.8%). One each of type IX (1.2%) and NT (1.2%) was identified among isolates. The genetic relatedness of GBS isolates was assessed by MLVA. By using a cutoff value of 85% similarity, a total of 15 different MLVA types (MTs) were identified among the isolates, and overall the diversity index obtained with this method was 0.84. The largest groups of similar MLVA profiles consisted of 16 (MT1, 19.3%) and 26 (MT8, 33.7%) isolates. Thirty five isolates were grouped in six MTs, one with four (MT2, 4.8%), eight (MT4, 9.6%), and seven (MT7, 8.4%) isolates each, and three with five (MTs 5, 6 and 13, 6.0%) isolates each. The other seven (8.4%) had unique MLVA profiles. Most GBS capsular type Ia were grouped in MT8 (23/35, 65.7%), and the other 12 isolates were distributed in seven distinct MLVA types. The GBS capsular types V and III were distributed in seven and three MLVA types respectively, whereas all isolates displaying the capsular type II were grouped in MT1, and all the isolates except one had an identical MLVA profile (Figure 1).

### Phenotypic and genotypic characterization of antimicrobial susceptibility

All isolates were susceptible to penicillin, ampicillin, cefepime, cefotaxime, chloramphenicol, levofloxacin and vancomycin. Resistance to erythromycin and clindamycin was detected in 16 (19.3%) and 11 (13.3%) isolates, respectively. All isolates resistant to clindamycin were also resistant to erythromycin, and among them only one had a constitutive macrolide-lincosamide-streptogramin B (cMLS_B_) phenotype (minimal inhibitory concentration - MIC > 8.0 *μ*g/mL for both antimicrobials) and harbored the *ermB* gene. Of the 10 isolates displaying the indutible MLS_B_ (iMLS_B_) phenotype, seven carried the *ermA* gene, whereas one isolate carried the *ermB* gene and two both genes. All isolates (*n* = 5) resistant only to erythromycin showed phenotype M and carried the *mefA/E* gene. Resistance to both erythromycin and clindamycin was detected among isolates belonging to serotypes V (*n* = 7) and III (*n* = 4), which were grouped in MTs 1, 3, 4, 6 and 7. All isolates resistant only to erythromycin belonged to serotype Ia and MT8 (Table 1).

**Table 1:** **Macrolide/lincosamide resistant *Streptococcus agalactiae*: distribution of capsular type, MLVA genotypes and antimicrobials resistance features**

**Isolate**	**Source**	**MLVA Genotypes**^**a**^	**Capsular type**^**b**^	**Erythromycin resistance phenotype**^**c**^	**Erythromycin resistance genes**^**d**^			**MIC (μg/mL)**^**e**^	
					***ermA***	***ermB***	***mefA/E***	**DA**	**E**
15	Urine	8	Ia	M	-	-	+	0.06	4.0
22	Urine	8	Ia	M	-	-	+	0.06	4.0
46	Urine	8	Ia	M	-	-	+	0.06	4.0
120	Urine	8	Ia	M	-	-	+	0.06	4.0
121	Swab	8	Ia	M	-	-	+	0.03	2.0
66	Urine	1	III	iMLS_B_	-	+	-	0.06	2.0
109	Urine	1	III	iMLS_B_	+	-	-	0.03	2.0
113	Urine	1	III	iMLS_B_	+	+	-	0.03	2.0
114	Urine	1	III	iMLS_B_	+	-	-	0.06	> 8.0
65	Urine	4	V	iMLS_B_	+	-	-	0.06	4.0
105	Urine	3	V	iMLS_B_	+	-	-	0.06	8.0
108	Urine	6	V	iMLS_B_	+	-	-	0.06	8.0
112	Urine	6	V	iMLS_B_	+	-	-	0.06	4.0
115	Swab	7	V	cMLS_B_	-	+	-	> 8.0	> 8.0
116	Swab	4	V	iMLS_B_	+	+	-	0.06	8.0
117	Urine	6	V	iMLS_B_	+	-	-	0.06	4.0

^a^The genetic diversity was assessed by MLVA typing [32]. A cutoff value of 85% similarity was applied to define MLVA types. ^b^The capsular type was identified by multiplex-PCR [43]. ^c^Erythromycin resistance phenotype was determined by the double-disk diffusion method [46]. ^d^The presence of specified gene was determined by PCR. (+) Presence; (-) Absence. ^e^The minimum inhibitory concentrations (MIC) were determined by the agar-dilution method. Clindamycin (DA); Erythromycin (E).

### Detection of potential virulence traits of commensal GBS

The presence of the nucleotide sequences corresponding to the *cylE*, *hylB and pilus* islands PI-1, PI-2a and PI-2b was detected by PCR. All isolates harbored the *cylE* and *hylB* genes and at least one *pilus* island. Four (4.8%) of the 83 GBS isolates did not produce a hemolytic halo around the bacterial colonies (Figure 1). Concomitantly, these isolates were not able to produce the orange carotenoid pigment in Granada medium. Most of the isolates harbored PI-2a alone (*n* = 30, 36.1%) or in combination with PI-1 (*n* = 42, 50.6%). PI-2a was distributed in all capsular types identified in this study, including the type IX and NT isolates. However, the presence of this *pilus* island alone or in combination with PI-1 was found mainly in capsular type Ia and V, respectively. Besides, PI-1 was also found in combination with PI-2b (*n* = 4, 4.8%) and all isolates belonged to capsular type III. The presence of PI-2b alone was observed in seven isolates (8.4%) and all belonged to capsular type Ia. All isolates grouped in MTs 1 (*n* = 16, 19.3%), 4 (*n* = 8, 9.6%), 6 (*n* = 5, 6.0%) and 7 (*n* = 7, 8.4%) harbored PI-1 and PI-2a islands. In addition, these *pili* were also detected in isolates belonged to MTs 2, 3, 5 and 15. All isolates belonging to MTs 8 (*n* = 26, 31.6%), 9, 10 and 11 (*n* = 1, 1.2% each) and one isolate (1.2%) of MT2 harbored the PI-2a island. PI-1 and PI-2b was detected only in isolates of MT5 (*n* = 4, 4.8%), whereas the PI-2b island was detected in isolates of MTs 12 (*n* = 1, 1.2%), 13(*n* = 5, 6.0%) and 14 (*n* = 1, 1.2%) (Figure 1). The isolates displaying the MLS_B_ phenotype harbored the *pilus* islands PI-1 and PI-2a, whereas the isolates showing the M phenotype harbored only the PI-2a.

## Discussion

In this study, five capsular types (Ia, II, III, V, IX) were identified and, except for type IX, all are frequently associated with GBS infections worldwide [3,7-9,20,21]. The serotypes identified in this study were also detected in different surveys that were performed with Brazilian isolates among pregnant and non-pregnant adults. In those studies, the serotypes Ib [10,11,31] IV [11,12], VI [10] and VIII [12] were also identified. The genetic diversity of GBS isolates were assessed by MLVA [32], which is based on the amplification of polymorphic tandem repeat sequences (also called VNTR-Variable Number of Tandem Repeats). It is easy to use, displays shorter time of execution, can be applied to a small or large numbers of isolates and has been employed successfully for the typing of different bacteria species. In addition, it has higher discriminatory power than Multi *Locus* Sequencing Typing (MLST), the reference method for genotyping *Streptococcus* spp. [32,33]. The diversity index obtained with MLVA for this bacterial population was 0.84, lower than observed by others [32,33]. However, despite the close relatedness of several isolates, as judged by the capsular type and presence of *pili* islands, this genotyping scheme discriminated the GBSs in this study. In fact, a total of 15 different genetic groups were identified among these isolates. SAG7, SAG21 and SAG22 presented higher degree of the variation in the number of repeats among the isolates, corroborating the data of Haguenoer et al. [32]. Although no statistical correlation was performed, it was observed that isolates belonging to the capsular type II were confined to MT1, indicating that the genetic background of this serotype may be well conserved. Higher number of isolates may corroborate these findings.

All isolates were susceptible to the antimicrobials evaluated in this study, except erythromycin and clindamycin. Although it was not an epidemiological investigation, the overall rate of erythromycin resistance among the isolates analyzed was 19.3%. Previous epidemiological and bacterial collection data from Brazilian GBS isolates showed that erythromycin resistance ranged from 4 to 14% [10-13]. A higher incidence rate was observed in other regions, where erythromycin resistance up to 40% among GBS isolates was detected in Europe [15] and USA [3,9]. In this study, resistance to both erythromycin and clindamycin was observed in GBS isolates of capsular types III and V, whereas the isolates displaying resistance only to erythromycin were exclusively found in the Ia capsular type. Similar results were previously obtained by other authors [3,10]; however, resistant isolates for both antimicrobials were also observed among the Ib, II, IV, VI and VIII capsular serotypes [3,34]. The mechanisms of macrolide resistance are mediated by *ermA*, *ermB* and *mefA/E,* and the distribution of these genes among GBS isolates in this study were in accordance with the macrolide-resistance phenotypes. These results were also observed by others [10-13]. The increasing numbers of isolates showing macrolide resistance together with the description of reduced susceptibility to penicillin emphasize the need for continued monitoring of antimicrobial susceptibility profile to identify the emergence of resistance among GBS isolates.

Data of the potential virulence of GBS isolates from Brazil are limited. Three genomic islands encoding the structurally distinct types of *pili* (PI-1, PI-2a and PI-2b) were identified in GBS. These *pili* are organized in two different *loci*, where PI-2a and PI-2b are located at the same chromosomal *locus*, with these being mutually exclusive [35]. To our knowledge, this is the first study describing the prevalence of the *pilus* island in Brazilian GBS isolates, and at least one *pilus* type was detected among the isolates, supporting their use as an antigen for vaccine development. The combination of PI-1 and PI-2a was the most prevalent among the GBS isolates, and this result is in agreement with previous reports [21,36]. In addition, the presence of this combination was correlated with maternal colonization and invasive disease in adults [36].

The *cyl locus* of GBS consists of a cluster of twelve genes [27], and some of them can modulate *cylE* expression and secretion [37], which is crucial for β-H/C activity. This toxin can promote the invasion of epithelial or endothelial cells [28], facilitate their persistence and intracellular survival [29], and enable their dissemination at host sites. Besides, acting as a virulence factor, CylE is associated with the characteristic translucent halo around GBS colonies grown on blood agar plates and production of orange carotenoid pigment on specific chromogenic agar, features that are used for presumptive identification of *S. agalactiae.* In this study, four GBS isolates were non-hemolytic and simultaneously non-pigment producers. Indeed, approximately 3% of GBS isolates are non-hemolytic [38], emphasizing the need to develop new methods that combine identification and detection of antimicrobial resistance for these bacteria.

The role of hyaluronidase in the pathogenesis of GBS infections is still unclear, but it is postulated that this enzyme can facilitate the invasion and dissemination of GBS during infection. The expression of this enzyme has been associated with GBS isolated from invasive infections [39]; however, hyaluronidase activity has also been detected in commensal GBS isolates from women’s genital tract [40].

## Conclusions

In conclusion, we identified the predominant occurrence of capsular types Ia, II, III and V among commensal GBSs isolated from women at reproductive age seen at University Hospital of Londrina, Paraná. The GBS isolates harbored at least one *pilus* island. Our findings are in agreement with a higher proportion of capsular types and distribution of *pili* previously reported among GBS isolated from different countries. These data support the notion of developing of a vaccine globally effective against this opportunistic bacterium. We also detected resistance to erythromycin and clindamycin and the occurrence of the genes encoding virulence determinants *cylE* and *hylB* among these isolates, reinforcing the need for continued monitoring of GBS to prevent the development of infections. In addition, a total of 15 different genetic groups were identified, and isolates belonging to the capsular type II were confined to MT1. Besides, resistance only to erythromycin was observed in GBS isolates belonging to capsular type Ia and MT8, whereas isolates resistant to both erythromycin and clindamycin were distributed over various capsular and MLVA types. Higher number of isolates may corroborate these findings.

## Methods

### Microorganisms

A total of 83 non-duplicate colonizing GBS isolates recovered from vaginal-rectal swabs (*n* = 31) and urine *(n* = 52) of women seen at University Hospital of Londrina, Paraná, Brazil from March to September of 2012 were randomly taken from the bacterial collection of the Laboratory of Clinical Microbiology of Universidade Estadual de Londrina. The isolates were classified according to CDC definitions of healthcare-associated infections [41]. Cultures were performed from the patients as part of the hospital surveillance study for healthcare-associated infections agents. All streptococci were identified to the species level by standard phenotypic methods on the basis of colony morphology, Gram staining, catalase and CAMP (Christie, Atkins, Munch-Petersen) tests, after growth on Muller-Hinton agar (MHA) containing 5% sheep blood at 37°C for 24 h. Concomitantly, tests for growth in 6.5% NaCl and in Granada™ Biphasic broth (Biomérieux), bile-esculin or sodium hippurate hydrolysis, and susceptibility to bacitracin and sulfamethoxazole plus trimethoprim were also performed. Bacteria were kept at -20°C in Tryptic Soy Broth (TSB, Oxoid) containing 20% glycerol and 5% sheep blood.

### DNA extraction

Total DNA of all GBS isolates was extracted following the procedures described by de-Paris *et al*. [42] with minor modifications. Briefly, a single bacterial colony was added to 3 mL TSB and incubated at 37°C for 24 h. The cultures were centrifuged at 10,000 x *g* for 5 min, the bacterial pellets were washed twice with sterile 0.15 M phosphate-buffered saline (PBS), pH 7.2, resuspended in 300 *μ*L sterile solution containing 10 mM Tris-HCl, 1 mM EDTA and boiled (100°C) for 20 min. Cellular debris was removed by centrifugation, and a 2-*μ*L aliquot of supernatant was used in all amplification reactions.

### Capsular typing and genotyping

The identification of capsular type (Ia, Ib, II-IX) of all GBS isolates was performed by multiplex PCR assay as described by Imperi *et al*. [43]. Non-typeable isolates were designated as NT.

The genetic clonal relatedness of the isolates was analyzed by MLVA using six markers named as SAG2, SAG3, SAG4, SAG7, SAG21 and SAG22 as described by Haguenoer *et al*. [32]. Cluster analysis were performed using the UPGMA algorithm of the Bionumerics v. 4.6 software (Applied Mathematics, Kortrijk, Belgium), and a cutoff value of 85% similarity was applied to define MLVA types. The genetic diversity in MLVA profiles of the isolates was calculated with Hunter-Gaston index [44].

### Antimicrobial susceptibility pattern

GBS isolates were tested for antimicrobial susceptibility to nine antimicrobials (ampicillin, cefepime, cefotaxime, chloramphenicol, clindamycin, erythromycin, levofloxacin, penicillin and vancomycin) using the disk-diffusion method. The minimum inhibitory concentrations (MIC) for erythromycin and clindamycin were determined by the agar-dilution method. MIC was determined at 100% growth inhibition. Both methods were performed and interpreted according to the Clinical Laboratory Standards Institute [45]. The GBS phenotypes showing resistance to erythromycin and clindamycin were determined by the double-disk diffusion method as described by Seppala *et al*. [46]. *Streptococcus pneumoniae* ATCC 49619 and *Enterococcus faecalis* ATCC 29212 were used as controls.

### PCR primer design and detection of virulence determinants and erythromycin and clindamycin resistance encoding genes

The nucleotide sequences of virulence determinants (*cylE*, *hylB* and *pilus* islands encoding PI-1, PI-2a and PI-2b) and erythromycin and clindamycin resistance (*ermA, ermB* and *mefA/E*) encoding genes from *S. agalactiae* deposited in the GenBank/EMBL databases were analyzed using the *BioEdit v.7.2.0* software. The sequences were aligned using *ClustalW* and a consensus sequence for each gene was used for specific primer design (Table 2). PCR was performed in a final volume of 25 *μ*L containing 20 mM Tris–HCl, pH 8.4, 5 mM KCl, 1.5 mM MgCl_2_, 100 *μ*M of each dNTP, 5 *p*mol of each forward and reverse primer, 2.5 U *Taq* DNA polymerase (Invitrogen, São Paulo, Brazil), and 2 *μ*L of genomic DNA. The amplification reactions were performed in a Veriti® 96-well Thermal Cycler (Applied Biosystems) with an initial denaturation at 95°C for 1 min, followed by 35 cycles of 95°C for 30 s, annealing at 60°C for 1 min and an extension step at 72°C for 45 s. Negative control reactions without any template DNA were carried out simultaneously. The identity of the amplicons was confirmed after determination of the nucleotide sequences with a 3730xl DNA Analyzer (Applied Biosystems) using the Big Dye® Terminator v.3.1 Cycle Sequencing Kit. Search for homologies in the GenBank/EMBL databases was carried out with the Blast algorithm.

**Table 2:** Description of primers used in PCR for the detection of virulence markers and erythromycin/clindamycin-resistance genes

**Target gene**^**a**^	**Sequence of the primer (5′ → 3′)**	**Amplicon size (bp)**	**Accession number**^**b**^
*hylB*	F: TGTCTCCGAGGTGACACTTGAACT	124	U15050.1/Y15903.1
	R: TTGTGTTGTGACGGGTTGTGGATG		
*cylE*	F: TCGGAACAAGTAAAGAGGGTTCGG	130	AF093787.2/AF157015.2
	R: GGGTTTCCACAGTTGCTTGAATGT		
PI-1	F: AACCACTAGCAGGCGTTGTCTTTG	147	EU929540.1/EU929469.1
	R: TGAGCCCGGAAATTCTGATATGCC		
PI-2a	F: GCCGTTAGATGTTGTCTTCGTACT	117	EU929374.1/EU929330.1
	R: TTTACTGCGGTCCCAAGAGCTTC		
PI-2b	F: AAGTCTTGACCAAGGATACGACGC	152	EU929426.1/EU929391.1
	R: ATCGTGTTACTTGCCCTGCGTA		
*ermA*	F: CCGGCAAGGAGAAGGTTATAATGA	190	EU492925.1/EU492926.1
	R: GCATTCACCCGTTGACTCATTTCC		
*ermB*	F: GCTCTTGCACACTCAAGTCTCGAT	117	EF422365.1/DQ250996.1
	R: ACATCTGTGGTATGGCGGGTAAGT		
*mefA/E*	F: GCGATGGTCTTGTCTATGGCTTCA	225	DQ445273.1/DQ445269.1
	R: AGCTGTTCCAATGCTACGGAT		

^a^*hylB,* hyaluronate lyase; *cylE*, hemolysin/cytolysin (β-H/C); PI-1, PI-2a, PI-2b, *pilus* islands; *ermA, ermB* cross-resistance to macrolides-lincosamide-streptogramin B; *mefA/E* resistance only to 14- and 15-membered ring macrolides. ^b^The nucleotide sequences of *Streptococcus agalactiae* genes deposited in the GenBank/EMBL databases used for specific primer design.

### Ethics statements

The study protocol was approved by the Ethics Committee of the Universidade Estadual de Londrina (Document 186/09-CEP/UEL). Written informed consent was obtained from the patients for the publication of this report and any accompanying images.

## Competing interests

The authors declare no competing interests.

## Authors’ contributions

E.S.O.: Contributed in all methodological activities and analysis and interpretation of data; A.E.B.M. and P.M.C.S.: Sample collection, identification of isolates and antimicrobial susceptibility assays; E.R.T. and A.T.M.: Nucleotide sequence analysis, primer design, amplicon sequencing; J.D.C.: MLVA analysis; L.M.Y. and M.R.E.P.: Interpretation of data and critical revision of the manuscript for important intellectual content. S.F.Y.O.: Conception, design, analysis and interpretation of data. All authors read and approved the final manuscript.
